# Steroidogenic factor-1 hypermethylation in maternal rat blood could serve as a biomarker for intrauterine growth retardation

**DOI:** 10.18632/oncotarget.21767

**Published:** 2017-10-10

**Authors:** Dong-Mei Wu, Liang-Peng Ma, Gui-Li Song, Yong Long, Han-Xiao Liu, Yang Liu, Jie Ping

**Affiliations:** ^1^ Department of Pharmacology, Wuhan University School of Basic Medical Sciences, Wuhan 430071, China; ^2^ Key Laboratory of Biodiversity and Conservation of Aquatic Organism, Institute of Hydrobiology, Chinese Academy of Sciences, Wuhan 430072, China; ^3^ Department of Pharmacy, Wuhan First Hospital, Wuhan 430022, Hubei, China

**Keywords:** steroidogenic factor-1 (SF-1), DNA methylation, intrauterine growth retardation (IUGR), clustered regularly interspaced short palindromic repeats/CRISPR-associated protein 9 (CRISPR/Cas9), biomarker

## Abstract

Intrauterine growth retardation (IUGR) is a common obstetric complication lacking an optimal method for prenatal screening. DNA methylation profile in maternal blood holds significant promise for prenatal screening. Here, we aimed to screen out potential IUGR biomarkers in maternal blood from the perspective of DNA methylation. The IUGR rat model was established by prenatal maternal undernutrition. High-throughput bisulfite sequencing of genomic DNA methylation followed by functional clustering analysis for differentially methylated region (DMR)-associated genes demonstrated that genes regulating transcription had the most significantly changed DNA methylation status in maternal blood with IUGR. Genes about apoptosis and placental development were also changed. Besides increased placental apoptosis, IUGR rats demonstrated the same hypermethylated CpG sites of steroidogenic factor-1 (SF-1, a DMR-associated transcription factor about placenta) promoter in maternal blood and placentae. Further, ff1b, the SF-1 ortholog, was knocked out in zebrafish by CRISPR/Cas9 technology. The knock-out zebrafish demonstrated developmental inhibition and increased IUGR rates, which confirmed the role of SF-1 in IUGR development. Finally, hypermethylated SF-1 was observed in human maternal blood of IUGR. This study firstly presented distinct DNA methylation profile in maternal blood of IUGR and showed hypermethylated SF-1 could be a potential IUGR biomarker in maternal rat blood.

## INTRODUCTION

Intrauterine growth retardation (IUGR), which refers to the poor fetal growth in uterus, is a common obstetric complication [1]. IUGR not only induces perinatal morbidity and mortality, but also increases the susceptibility to some age-related diseases in the offspring, such as hypertension and type II diabetes [2–4]. Owing to the lack of effective intrauterine treatments, the current goal of IUGR management is early detection and timely delivery [5, 6]. Multiple methods have been combined for IUGR prediction, including biophysical profile, routine ultrasound, and molecular biological technique [7]. But there still lacks a definitively optimal method for screening IUGR during gestation.

The etiology of IUGR is largely attributed to adverse intrauterine environment during pregnancy. Epigenetics explains the relationship between gene expression and environmental signals. As a major mechanism of epigenetic modification, DNA methylation can affect the stable maintenance of gene expression patterns [8, 9]. Meanwhile, DNA methylation is more sensitive to environmental stimuli than DNA sequences and more stable than RNA in maternal blood [8, 10]. Maternal blood provides gas and nutrients for the developing fetus and could provide an avenue of signal exchange [7]. DNA methylation analysis in maternal blood has been considered as a non-invasive method with advantages of good accuracy and sensitivity. It has been reported that DNA methylation patterns in maternal blood could be used for non-invasive prenatal diagnosis of trisomy 21 and congenital heart defects [11, 12]. As to prenatal IUGR screening, hypermethylated RASSF1A in maternal blood was reported to be a possible IUGR biomarker [13]. It remains unclear whether genomic DNA methylation profile was generally changed in maternal blood of IUGR. And more evidence is needed to verify the role of aberrant DNA dysfunction in IUGR.

The placenta serves as an essential barrier for fetal development. Placental dysfunction-induced adverse intrauterine environment is taken as one of the predominant causes of IUGR [6, 14, 15]. And the underlying mechanism may be associated with the aberrant epigenetic modification in the placenta [16, 17]. steroidogenic factor-1 (SF-1) is an IUGR-related transcription factor crucial for placental development and syncytiotrophoblast apoptosis, of which the expression is associated with its promoter methylation status [18–21]. As apoptotic syncytiotrophoblast cells flow into the maternal blood, placental DNA can be determined in the maternal blood [22, 23]. Thus, the aberrant DNA methylation of genes in IUGR placenta (such as SF-1) and the subsequent placental dysfunction may be reflected by the DNA methylation patterns in maternal blood.

In this study, we aimed to demonstrate DNA methylation profile in maternal blood of IUGR and to screen out potential IUGR biomarkers. The IUGR rat model was established by maternal undernutrition during pregnancy. And we used high-throughput sequencing of genomic DNA methylation to screen out changed DNA status of genes related to fetal and placental development in maternal blood. The screen-out gene was knocked out in zebrafish to verify its role in inducing IUGR. Further, the DNA methylation pattern of the screen-out gene was confirmed in peripheral blood of clinical pregnant women with IUGR. This study will provide evidence for understanding IUGR pathophysiology and the non-invasive prenatal prediction of IUGR.

## RESULTS

### Developmental parameters in rats

**Figure 1:**
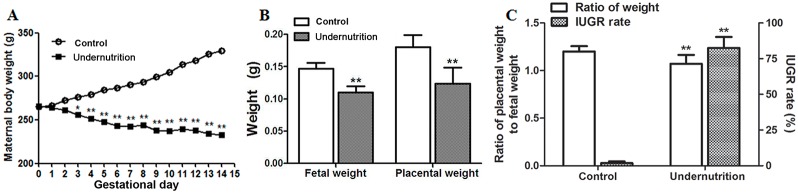
Developmental parameters of rats under prenatal maternal undernutrition The pregnant rats were fed a restricted diet (50% of the daily food intake of the control rats) from gestational day (GD) 0 to GD14. On GD14, the rats were anesthetized with isoflurane and sacrificed. **(A)** Maternal body weights; **(B)** fetal weights and placental weights; **(C)** intrauterine growth retardation (IUGR) rates and the ratio of placental weight to fetal weight and. Mean ± SD, ^*^P<0.05, ^**^P<0.01, two-sided *t*-test, *n* = 6 control *vs.* 5 undernutrition pregnant rats.

In this study, an IUGR rat model was established successfully through prenatal maternal undernutrition. As shown in Figure 1A, maternal body weights of the control gained gradually during gestation. However, maternal body weights in the undernutrition group decreased during gestation and were significantly lower than that of the control since gestational day (GD) 3. On GD14, fetal body weights and placental weights in the undernutrition group were reduced to 75.0% and 68.8%, respectively, of the control (*P*<0.01, Figure 1B). And the ratio of placental weight to fetal weight in the undernutrition group was significantly lower than that in the control (*P*<0.01, Figure 1C). IUGR rate in the undernutrition group increased to 82.5%, which was 44 times higher than that in the control (*P*<0.01, Figure 1C).

### Genomic DNA methylation sequencing in maternal rat blood

High-throughput genomic DNA methylation sequencing was employed to demonstrate DNA methylation profile in maternal rat blood. The majority of differentially methylated regions (DMRs) were distributed within intergenic regions, including promoters. The functional clustering analysis for top 3000 DMR-associated genes demonstrated that the most significantly changed DNA methylation patterns were in genes regulating transcription (Table 1). Placenta-related transcription factors of this cluster were shown in Table 2. Among the functional clusters related to fetal and placental development, remarkable changes were observed in apoptosis, cardiac development, neurodevelopment and placental development (Table 1). The specific genes involved in these functional clusters were shown in Table 2.

**Table 1:** Functional clusters for DMR associated genes

GO term	Count	*P* value	FDR
**Transcription regulation**	**Enrichment score: 3.46**		
GO:0030528~transcription regulator activity	174	0.001	2.04
GO:0003677~DNA binding	238	0.009	13.46
GO:0006350~transcription	234	0.020	30.88
GO:0003700~transcription factor activity	108	0.030	38.42
GO:0045449~regulation of transcription	285	0.039	52.03
**Apoptosis**	**Enrichment Score: 2.30**		
GO:0008219~cell death	80	0.004	7.12
GO:0016265~death	81	0.005	8.88
GO:0006915~apoptosis	73	0.007	11.79
GO:0012501~programmed cell death	73	0.010	17.05
**Cardiac development**	**Enrichment Score: 1.48**		
GO:0060415~muscle tissue morphogenesis	8	0.003	5.11
GO:0055008~cardiac muscle tissue morphogenesis	8	0.003	5.11
GO:0003007~heart morphogenesis	18	0.004	7.59
GO:0055010~ventricular cardiac muscle morphogenesis	7	0.005	8.58
GO:0048738~cardiac muscle tissue development	14	0.012	20.53
**Neurodevelopment**	**Enrichment Score: 1.40**		
GO:0030030~cell projection organization	53	0.007	12.78
GO:0000904~cell morphogenesis involved in differentiation	37	0.013	20.93
GO:0032989~cellular component morphogenesis	54	0.028	40.93
GO:0048666~neuron development	46	0.030	42.73
GO:0048667~cell morphogenesis involved in neuron differentiation	31	0.031	43.92
GO:0030182~neuron differentiation	60	0.031	44.41
GO:0048812~neuron projection morphogenesis	30	0.034	46.66
GO:0007409~axonogenesis	28	0.037	50.05
**Placental development**	**Enrichment Score: 0.86**		
GO:0060716~labyrinthine layer blood vessel development	5	0.031	74.02
GO:0001890~placenta development	15	0.041	92.43

DMR, differentially methylated region; FDR, false discovery rate.

**Table 2:** Genes involved in functional clusters for DMR associated genes

Gene symbol	DMR_ID	Description	Methylation status	Fold change
**Genes about transcription regulation**				
Cdx2	Chr12_dmr_912	caudal type homeo box 2	hypermethylated	2.99
Cry2	Chr3_dmr_9973	cryptochrome circadian clock 2	hypermethylated	2.67
Rara	Chr10_dmr_13437	retinoic acid receptor alpha	hypermethylated	3.12
SF-1	Chr3_dmr_2513	steroidogenic factor-1	hypermethylated	3.67
**Genes about apoptosis**				
Steap3	Chr13_dmr_2602	STEAP family member 3, metalloreductase	hypomethylated	-2.01
Nfkb1	Chr2_dmr_26888	nuclear factor of kappa light polypeptide gene enhancer in B-cells 1	hypomethylated	-2.56
Inha	Chr9_dmr_10674	inhibin alpha	hypomethylated	-3.09
**Genes about cardiac development**				
Tbx1	Chr11_dmr_11267	T-box 1	hypermethylated	3.21
Foxc2	Chr19_dmr_7361	forkhead box C2	hypermethylated	2.42
Pitx2	Chr2_dmr_26037	paired-like homeodomain 2	hypermethylated	2.54
**Genes about neurodevelopment**				
Cdk5	Chr4_dmr_868	cyclin-dependent kinase 5	hypomethylated	3.42
Lhx4	Chr13_dmr_7436	LIM homeobox 4	hypermethylated	2.89
**Genes about placental development**				
Ncoa6	Chr3_dmr_19379	nuclear receptor coactivator 6	hypermethylated	2.78
Ovol2	Chr3_dmr_17597	ovo-like zinc finger 2	hypermethylated	3.21

DMR, differentially methylated region.

### DNA methylation status of SF-1 promoter in maternal rat blood

**Figure 2:**
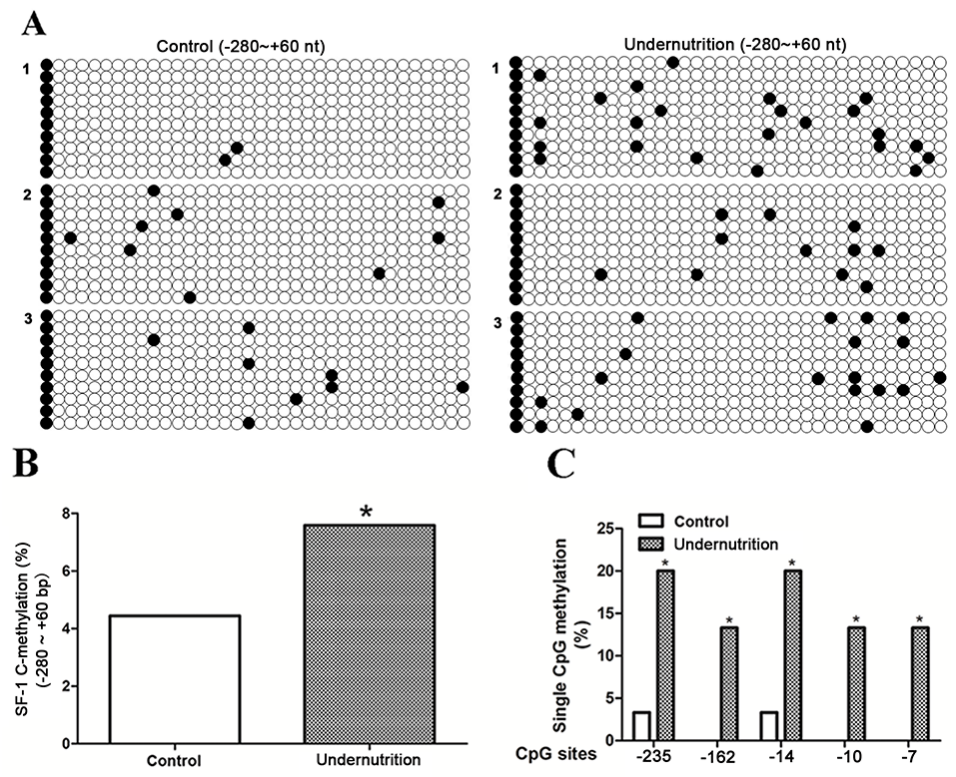
DNA methylation status of steroidogenic factor 1 (SF-1) in maternal rat blood under prenatal maternal undernutrition CpG methylation status in the -280/+60 domain of rat SF-1 was detected by the bisulfite sequencing PCR (BSP) method. +1 is the transcription start site. **(A)** The methylation map. Black and white circles represent methylated and unmethylated CpGs, respectively. **(B)** The total methylation rate of CpG sites; **(C)** the CpG sites with changed methylation frequency. ^*^P<0.05, Fisher's exact test, *n* = 3 pregnant rats.

Among the 4 differentially methylated transcription factor, SF-1 has been proven as an IUGR-related gene in our previous studies [20]. Meanwhile, SF-1 plays an important role in placental development and syncytiotrophoblast apoptosis, another two changed functional clusters [18, 19, 21]. Thus, hypermethylated SF-1 was selected for further investigation. It is widely accepted that hypermethylated gene promoter could induce inhibited gene expression via disturbing the binding with transcription factors [10, 24]. Thus, the methylation status of CpG sites in the -280/+60 domain of rat SF-1, which covers the binding sites for transcription factors, was verified in maternal blood. Figure 2A demonstrated the methylation map of CpG sites. As shown in Figure 2B, the total methylation rate in the undernutrition group was significantly higher than that in the control (*P*<0.05). Among the 36 CpG sites, nt -235, -162, -14, -10 and -7 showed increased frequency of single CpG methylation after undernutrition treatment (*P*<0.05, Figure 2C).

### Placental histology and apoptosis in rats

**Figure 3:**
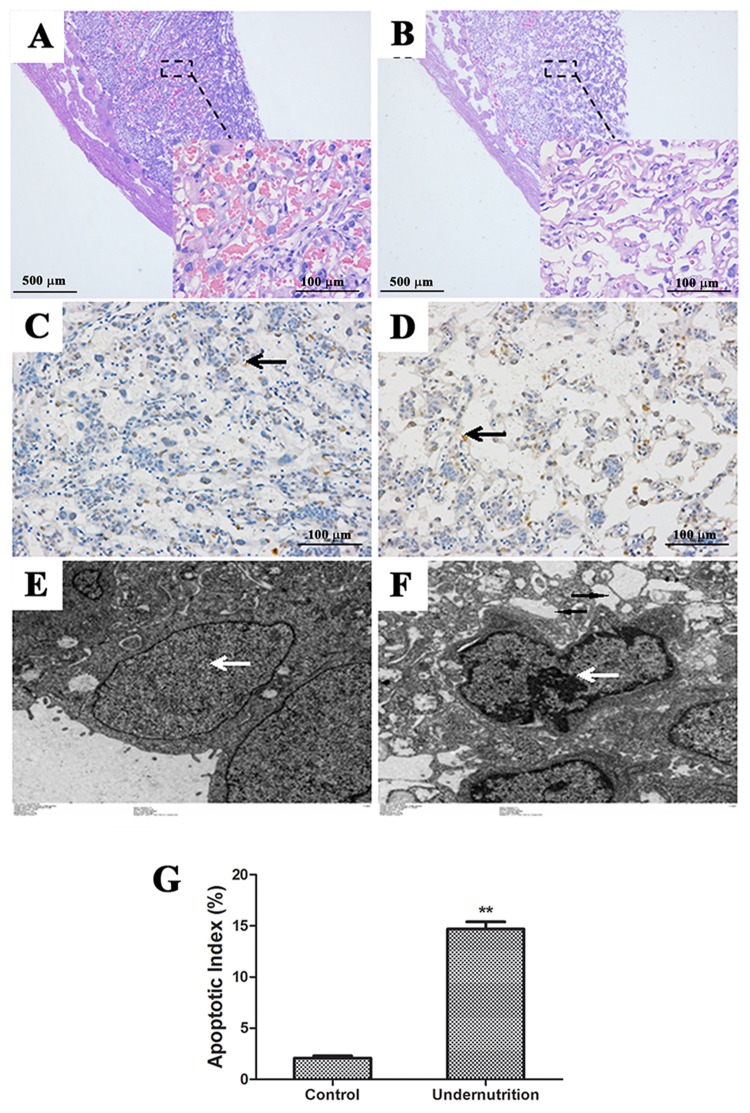
Histological changes and cell apoptosis in rat placentae under prenatal maternal undernutrition Paraffin sections stained with hematoxylin and eosin (HE) were observed under a light microscope. **(A)** Control (40×, 200×); **(B)** undernutrition (40×, 200×). Syncytiotrophoblast apoptosis in the labyrinth zone was detected by TdT-mediated dUTP nick end labeling (TUNEL). **(C)** Control (200×); **(D)** undernutrition (200×). Black arrow: syncytiotrophablast. The submicroscopic structure of syncytiotrophoblast was analyzed by transmission electron microscope (TEM). **(E)** Control (2000×); **(F)** undernutrition (2000×). White arrow: nucleus. Black arrow: vacuoles. **(G)** Semi-quantitative analysis of TUNEL. Mean ± SD, ^*^P<0.05, two-sided *t*-test, *n* = 3 pregnant rats.

Increased placental apoptosis was reported to induce aberrant placental development and IUGR [25, 26]. Thus, we analyzed the histology and cell apoptosis in rat placentae. As compared to the control (Figure 3A), fewer syncytiotrophoblasts and more dilated intercellular spaces were shown in the IUGR placentae stained with hematoxylin and eosin (HE) (Figure 3B). In addition, TdT-mediated dUTP nick end labelling (TUNEL) staining showed increased apoptosis in syncytiotrophoblasts of IUGR placentae (Figure 3D) than that of the control (Figure 3C). The data from the semi-quantitative assay also demonstrated a statistically significant difference between the two groups (*P*<0.01, Figure 3G). The same phenomenon was also observed by transmission electron microscope (TEM) analysis, which exhibited typical apoptotic morphological changes in placental syncytiotrophoblasts of the IUGR group (Figure 3F) but not in the control (Figure 3E). Specifically, these shrunken syncytiotrophoblast cells had intense chromatin structure at the periphery of nucleus (white arrows) and had cytoplasm rich in vacuoles (black arrows).

### mRNA expression and DNA methylation of SF-1 in rat placentae

**Figure 4:**
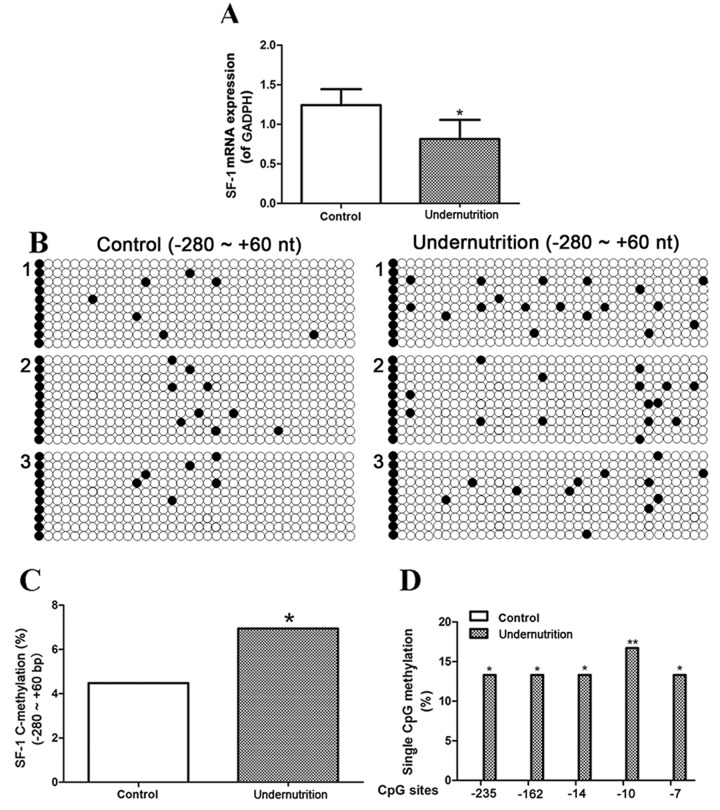
mRNA expression and DNA methylation of steroidogenic factor 1 (SF-1) in rat placenta under prenatal maternal undernutrition **(A)** Real-time quantitative PCR was used to detect the mRNA expression. Mean ± SD, ^*^P<0.05, two-sided *t*-test, *n* = 6 control vs. 5 undernutrition pregnant rats. CpG methylation status in the -280/+60 domain of rat SF-1 was detected by the bisulfite sequencing PCR (BSP) method. +1 is the transcription start site. **(B)** The methylation map. Black and white circles represent methylated and unmethylated CpGs, respectively. **(C)** The total methylation rate of CpG sites; **(D)** the CpG sites with changed methylation frequency. ^*^P<0.05, ^**^P<0.01, Fisher's exact test, *n* = 3 pregnant rats.

To explore whether the changed DNA methylation of SF-1 also occurred in placentae, we determined the DNA methylation and mRNA expression of SF-1 in rat placentae. The reverse-transcription PCR (RT-PCR) analysis exhibited that SF-1 mRNA expression in the undernutrition placentae markedly reduced to 65.5% of the control (*P*<0.05, Figure 4A). Figure 4B demonstrated the methylation map of CpG sites in the -280/+60 domain of rat SF-1. The total methylation rate of SF-1 promoter in the undernutrition group was significantly higher than that in the control (*P*<0.05, Figure 4C). More importantly, the hypermethylated SF-1 CpG sites in rat placentae were the same as those in maternal blood under prenatal maternal undernutrition (*P*<0.05, Figure 4D).

### Developmental parameters and mRNA expression of developmental genes in fushi tarazu factor 1b (ff1b) knockout zebrafish

**Figure 5:**
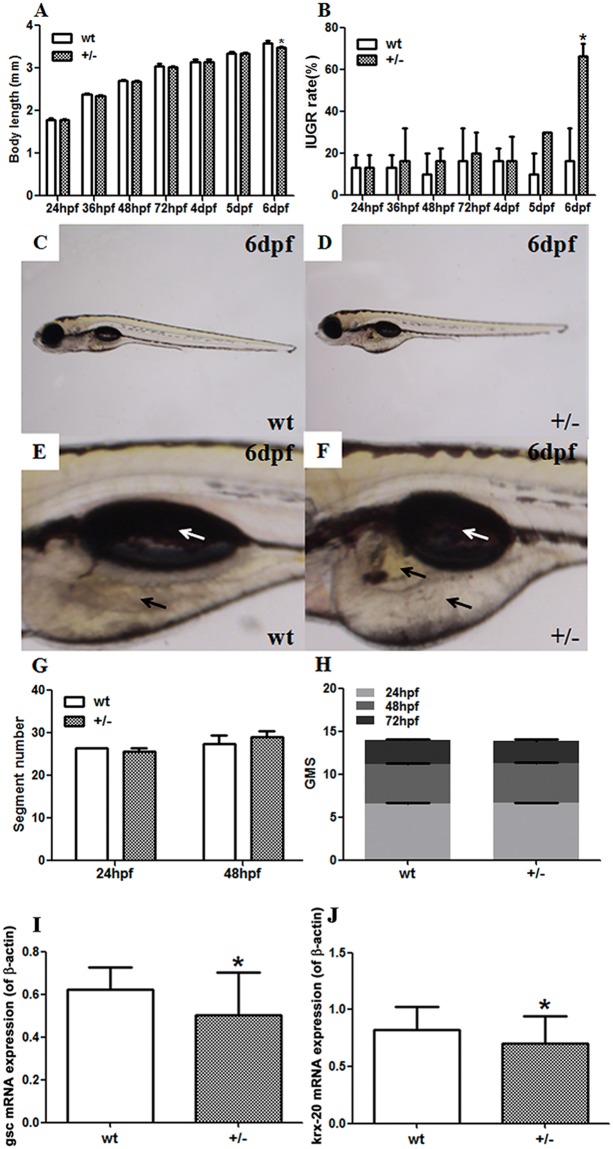
Developmental parameters and mRNA expression of developmental genes in fushi tarazu factor 1b (ff1b) knockout zebrafish by CRISPR/Cas9 technology (A-D) Developmental parameters of F_3_ zebrafish embryos; **(A)** body lengths; **(B)** intrauterine growth retardation (IUGR) rates; **(C)** the whole body of F_3_ wild-type (WT) embryos at 6 days post-fertilization (dpf); **(D)** the whole body of F_3_ mutant-type (MT) embryos at 6 dpf; **(E)** the yolk and swim bladder of F_3_ WT embryos at 6 dpf; **(F)** the yolk and swim bladder of F_3_ MT embryos at 6 dpf. White arrow: swim bladder; black arrow: yolk. **(G)** Segment number; **(H)** general morphology score (GMS). Mean ± SD, ^*^P<0.05, two-sided *t*-test, *n* = 3 F_2_ zebrafish. Real-time reverse-transcription PCR (RT-PCR) was used to detect the mRNA expression of developmental genes in F_3_ embryos. **(I)** goosecoid (gsc) expression in F_3_ embryos at 12 hours post-fertilization (hpf); **(J)** krox20 expression in F_3_ embryos at 12 hpf. Mean ± SD, ^*^P<0.05, two-sided *t*-test, *n* = 3 embryos.

The SF-1 ortholog ff1b, was knocked out in zebrafish by clustered regularly interspaced short palindromic repeats/CRISPR-associated protein 9 (CRISPR/Cas9) technology (Supplementary Figure 2). Developmental parameters of F_3_ zebrafish were measured to analyze the effects of ff1b on zebrafish development. There were significantly decreased body lengths (*P*<0.05, Figure 5A) and increased IUGR rates (*P*<0.05, Figure 5B) in mutant-type (MT) embryos at 6 days post-fertilization (dpf) in comparison to the wild-type (WT) embryos. Different from the WT group (Figure 5C, 5E), the 6-dpf MT embryos demonstrated delayed yolk sac absorption, accompanying with yolk sac edema and smaller fish bladders (Figure 5D, 5F). The body segment numbers (Figure 5G) and general morphology score (GMS) (Figure 5H) showed no significant changes at the observed time points. To evaluate the delayed embryonic development at the organogenic level, the expression of goosecoid (gsc) and krx-20 were determined in F_3_ zebrafish embryos at 12 hour post-fertilization (hpf). Both mRNA levels of gsc (*P*<0.05, Figure 5I) and krx-20 (*P*<0.05, Figure 5J) significantly decreased in F_3_ MT zebrafish embryos compared to the WT group.

### DNA methylation of SF-1 promoter in human maternal blood

**Figure 6:**
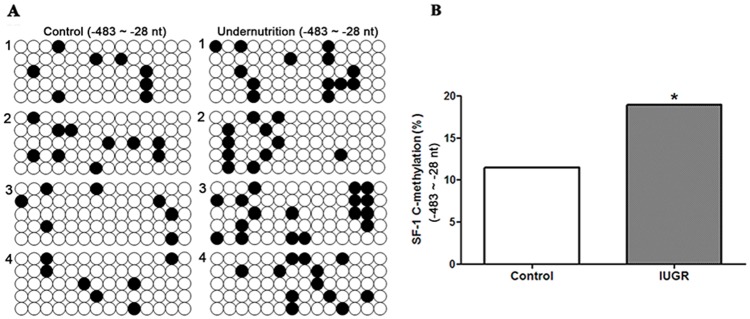
DNA methylation status of steroidogenic factor 1 (SF-1) in human maternal blood of normal pregnancies and pregnancies with intrauterine growth retardation (IUGR) CpG methylation status in the -483/-28 domain of human SF-1 was detected by the bisulfite sequencing PCR (BSP) method. +1 is the transcription start site. **(A)** The methylation map. Black and white circles represent methylated and unmethylated CpGs, respectively. **(B)** The total methylation rate of CpG sites. ^*^P<0.05, Fisher's exact test, *n* = 4 pregnant women.

Further, we collected human maternal blood to confirm the DNA methylation status of SF-1 promoter in normal and IUGR pregnancies. The clinical characteristics of pregnant women were presented in Table 3. There were no intergroup differences in maternal age, gestational age (GA) at delivery and body mass index (BMI). The birth weight was significantly lower in IUGR groups compared with the control group (*P*<0.05). The methylation status of CpG sites within the -483/-28 domain of SF-1 in maternal blood, which covers the binding sites for transcription factors, was detected by bisulfite sequencing PCR (BSP) analysis. Figure 6A exhibited the methylation map of CpG rich region in the two groups. The increased total methylation rates of SF-1 promoter were observed in IUGR pregnant women (*P*<0.05, Figure 6B). Few differences at each single CpG site were shown between the two groups.

**Table 3:** Clinical characteristics of pregnant women

Characteristic	Controls (*n*=4)	IUGR (*n*=4)
Maternal age (y)	31.7 ± 3.2	32.2 ± 3.5
BMI (kg/m^2^)	19.8 ± 2.9	22.1 ± 3.0
Primigravida (n)	4	4
GA at delivery (wk)	39.3 ± 1.1	38.9 ± 1.8
Birth weight (kg)	3.4 ± 0.4	2.1 ± 0.3^*^

Data were evaluated with two-sided *t*-test, and were given as mean ± SD and number. ^*^P<0.05 vs control. BMI, body mass index; GA, gestational age; IUGR, intrauterine growth restriction.

## DISCUSSION

### DNA methylation patterns in maternal blood may reflect increased placental apoptosis in IUGR

Prenatal maternal undernutrition is the most common method to establish IUGR models with a high IUGR rate. Based on our previous studies [27], the IUGR rat model was successfully established via a restricted diet from the early to the middle gestation. According to the results of genomic DNA sequencing, maternal blood of the undernutrition group demonstrated changed DNA methylation status of genes related to transcription, apoptosis, cardiac development, neurodevelopment and placental development. These results were in line with our previous studies on nicotine-induced IUGR [28]. Similar results were also found in placentae with early-onset pre-eclampsia by Blair et al [8]. Cardiovascular system and nervous system are two vital systems for fetal development. It has been reported that symmetric IUGR is associated with the development of cardiovascular disease, coronary heart disease and a relatively larger brain [29, 30]. Damaged placental development and increased apoptosis can inhibit the fetal development through inducing an adverse intrauterine environment [6, 14, 31]. Therefore, we proposed that the above-mentioned DNA methylation changes in maternal blood may reflect the occurrence of IUGR.

Among these changed functional clusters, increased placental apoptosis has been reported to be a possible mechanism for placental dysfunction and the occurrence of IUGR [25, 26]. Meanwhile, excessive placental trophoblast apoptosis has been reported to be reflected by extracellular DNA hypermethylation in maternal plasma [32]. Our previous study has demonstrated that caffeine treatment in rats may induce IUGR by increasing p53-dependent placental apoptosis [31]. In our present study, placentae were sampled on GD14, when the placenta is definitively formed [33]. Besides the relatively lower placental weights, the histological damages were observed in HE-staining IUGR placentae. Furthermore, the increased numbers of apoptotic cells was observed in the IUGR placentae using TUNEL and TEM detection. Thus, we speculated that increased placental apoptosis in IUGR may be reflected by the DNA methylation patterns in maternal blood.

### Hypermethylated SF-1 in maternal blood could reflect its hypermethylation and increased apoptosis in IUGR placentae

In this high-throughput sequencing, genes regulating transcription demonstrated the most significantly changed DNA methylation status. And the 4 DMR-associated transcription factors about placenta are crucial for maintaining placental function. Caudal type homeo box 2 (CDX2) enhances trophoblast cell invasion by altering the expression of matrix metalloproteinases [34]. Cryptochrome circadian clock 2 (CRY2) regulates the normal placental phenotype by affecting physiological rhythms [35]. Retinoic acid receptor alpha (RARA) regulates placental steroidogenesis by mediating mRNA expression and enzymatic activity of 17-beta hydroxysteroid dehydrogenase type 2 (HSD17B2) [36]. SF-1 plays an important role in placental morphogenesis. Aberrant DNA methylation and expression of SF-1 can lead to placental apoptosis and IUGR [21, 37]. Meanwhile, SF-1 methylation has been explored in the development of endocrine organs [38]. Inhibited SF-1 expression was observed on GD20 in fetal adrenals of IUGR rats induced by different prenatal exposures [20, 39]. Huang et al. [40] also observed inhibited SF-1 expression in adrenals of adult IUGR rats. In addition, we exposed zebrafish embryos to different IUGR risk factors (caffeine and nicotine) and observed lower ff1b expression and lower body length since 48hpf (Supplementary Figure 1). These reports imply that IUGR occurrence may be accompanied by persistently inhibited SF-1 expression. As to maternal blood, notably hypermethylated SF-1 was observed in our previous prenatal nicotine-induced IUGR rats [28]. Combing our and others’ results, we chose SF-1 DNA methylation for further verification as a potential biomarker for IUGR in maternal blood.

The DNA methylation pattern of peripheral blood, which can reflect the global DNA methylation patterns, has been reported to predict the changed DNA methylation pattern occurring in the specific tissue [41]. In this study, BSP analysis confirmed the increased SF-1 methylation in both maternal blood and placentae of the undernutrition group. More importantly, the significantly changed 5 CpG sites in maternal blood were the same as those in the placentae. Thus, DNA methylation status of SF-1 in maternal blood could reflect that in the placenta. Decreased SF-1 mRNA expression was also observed in placentae of IUGR, which was in line with the effects of hypermethylated SF-1 on the corresponding mRNA expression. SF-1 was reported to affect placental apoptosis by regulating the expression of pro-apoptotic genes [19, 21, 37]. Meanwhile, increased placental apoptosis was reported to be reflected in the maternal blood. For example, apoptotic trophoblast cells may release IUGR-related mRNA into the maternal blood, which could be determined to predict IUGR [5, 6]. By culturing the primary trophoblastic cells, Tjoa et al. [23] observed the relationship between the increased cell-free DNA in the supernatant and the increased cell apoptosis. Thus, we speculated that hypermethylated SF-1, which can inhibit SF-1 expression and increase placental apoptosis, may be reflected in maternal blood and as a potential IUGR biomarker.

### Hypermethylated SF-1 in maternal blood could be a potential IUGR biomarker

Further we aimed to verify the role of DNA methylation of SF-1 in fetal development. At present, the modification of DNA methylation in the specific region of a single gene has not been conducted successfully *in vivo*. But it is well-known that DNA hypermethylation decreases gene expression at the level of transcription. Therefore, we knocked out maternal SF-1 expression to represent DNA hypermethylation status of SF-1, and investigated the effects of knock-out SF-1 on the embryonic development to verify the relationship between increased DNA methylation of SF-1 and IUGR. Due to the shorter experimental period and the higher mutation rate, ff1b (the SF-1 ortholog in zebrafish) was knocked out in zebrafish by CRISPR/Cas9 technology. The mutant zebrafish were bred for three generations to minimize the off-target effects of the genome editing.

In this study, we successfully developed hereditable ff1b knockout MT zebrafish, which can be reflected by the decreased ff1b mRNA expression in F_2_ MT zebrafish (Supplementary Figure 2). The mutant homozygotes in our study can only survive up to 15 dpf, which is in line with the report by Chai et al [42]. Thus, mutant heterozygotes were used for the subsequent experiments. The results showed that the F_3_ MT embryos had inhibited developmental parameters and increased IUGR rates at 6 dpf, which is equivalent to GD18.5 in mice [43]. At the genetic level, we also observed the significantly reduced expression of two ozganogenetic biomarkers (gsc and krx-20) in 12-hpf MT embryos [44, 45]. Thus, the knockout of maternal SF-1 could inhibit the expression of developmental genes since the early prenatal phase when the phenotypes are still inconspicuous. The delayed phenotypic changes may be resulted from the use of heterozygotes. These results demonstrated that decreased SF-1 expression can cause developmental inhibition and IUGR.

Furthermore, we compared the DNA methylation status of SF-1 promoter in human maternal blood between normal pregnancy and pregnancy with IUGR. In order to reduce sampling deviation, age, BMI and gestational age were kept consistent between the two groups. Although no single CpG sites of the SF-1 promoter showed significantly different DNA methylation rates between the two groups, the total methylation frequency of SF-1 promoter in the IUGR group was much higher than that of the control. These results implied the possibility of SF-1 hypermethylation in maternal blood as a potential IUGR biomarker in clinic, despite the relatively low number of pregnant women in this study.

## MATERIALS AND METHODS

### Chemicals and reagents

Isoflurane was obtained from Baxter Healthcare Co. (Deerfield, IL, USA). The TUNEL kit was from Promega Co. (Madison, WI, USA). The genomic DNA preparation kit was purchased from Vitagene Co. (Hangzhou, China). The pGEM-T Easy vector was from Promega Co. (Madison, WI, USA). T7 endonuclease I (T7EI), pXT 7 vector and pMD 19-T vector were purchased from New England Biolabs Inc. (Beverly, MA, USA). The mMESSAGE mMACHINE kit was from Ambion (Austin, TX, USA). The TIANgel Midi purification kit was obtained from Tiangen Biotech Co., Ltd. (Beijing, China). The EZ DNA methylation kit was provided by Zymo Research Co. (Orange, CA, USA). All other chemicals and reagents were of analytical grade.

### Experiments in rats

#### Animals and treatments

Specific pathogen-free Wistar rats, females weighing 200 ± 20 g and males weighing 280 ± 20 g were from the Experimental Center of the Hubei Medical Scientific Academy (No. 2008-0005, Hubei, China). The rat experiments were performed in the Center for Animal Experimentation of Wuhan University (Wuhan, Hubei, China), which has been accredited by the Association for Assessment and Accreditation of Laboratory Animal Care (AAALAC) International.

Two female rats were mated with one male rat overnight after 7 days of acclimation. The day to observe vaginal smear with sperm cells was designated as GD0. Rats in the control group were allowed free access to standard chow and water, while rats in the undernutrition group were put on a restricted diet (50% food intake of the GD-matched control rats) from GD0. On GD14, pregnant rats were anesthetized with isoflurane and euthanized by decapitation. Maternal blood was collected in sodium citrate tubes. Fetuses and placentae were weighed after being dried on filter paper. IUGR was diagnosed when a fetal weight was two standard deviations lower than the mean body weight of control group [46]. IUGR rates were calculated by dividing the number of IUGR in each litter by the total pups of the litter. Three placentae in each group were randomly selected for fixing in 10% neutral formalin solution to make paraffin slices. Another three placentae in each group were randomly selected and immersed in 3% glutaraldehyde/1.5% paraformaldehyde solution for TEM analysis. The remaining placentae were stored at -80°C.

#### Histopathological measurement of placentae

For light microscopy analysis, 5 μm-thick paraffin sections were stained with HE and TUNEL, respectively. For TEM analysis, samples were postfixed for 1.5 h in 1% osmium tetroxide/1.5% potassium ferrocyanide solution, dehydrated in graded concentrations of ethanol, and embedded in Epon 618. Ultrathin sections approximately 70 nm thick were stained with uranyl acetate and lead citrate, and examined using a Hitachi H600 TEM (Hitachi, Co., Tokyo, Japan).

#### High-throughput bisulfite sequencing in maternal blood

Genomic DNA was isolated from 3 samples of maternal blood using the genomic DNA preparation kit. 5 ng of genomic DNA was fragmented into 400-600 nt using M220 Focused-ultrasonicator from Covaris Inc. (Woburn, MA, USA), followed by bisulfite conversion with the EZ DNA methylation kit. Bisulfite-converted DNA was used for illumina pair-end library preparation with the EpiGonme Methyl-seq kit. The libraries were applied to illumine Nextseq 500 system for 150 nt pair-end high-throughput sequencing by ABlife Inc. (Wuhan, Hubei, China). DMRs were identified based on the DNA methylation status of every sample. Among the top 3000 DMR associated genes, functional clustering analysis was employed to screen out the functional clusters and genes needing subsequent confirmation.

#### BSP analysis in maternal blood and placentae

The methylation status of CpG sites in the -280/+60 domain of SF-1 proximal promoter was determined by BSP analysis. The DNA sequence of this domain was referred to UCSC Genome Browser (http://genome.ucsc.edu/) and demonstrated in Supplementary Database 1. 10 clones were sequenced for each sample. The detailed protocols were published in our previous study [20].

#### Real-time RT-PCR analysis in placentae

Placental SF-1 mRNA expression was determined by real-time RT-PCR. Detailed protocols were published in our previous study [20].

### Experiments in zebrafish

#### Maintenance of zebrafish

WT AB strain zebrafish were supplied by the Key Laboratory of Biodiversity and Conservation of Aquatic Organism, Institute of Hydrobiology, Chinese Academy of Sciences (Wuhan, Hubei, China). Zebrafish were raised at 28 ± 0.5 °C in a 14:10 h light: dark cycle in a closed flow-through system. Zebrafish embryos were obtained from spawning adults in groups of 20 males and 10 females in tanks overnight. Spawning was induced in the morning when the light was turned on. Normally developed embryos were selected at 4-5 hpf.

#### CRISPR/Cas9 targeted mutation technology

**Figure 7:**
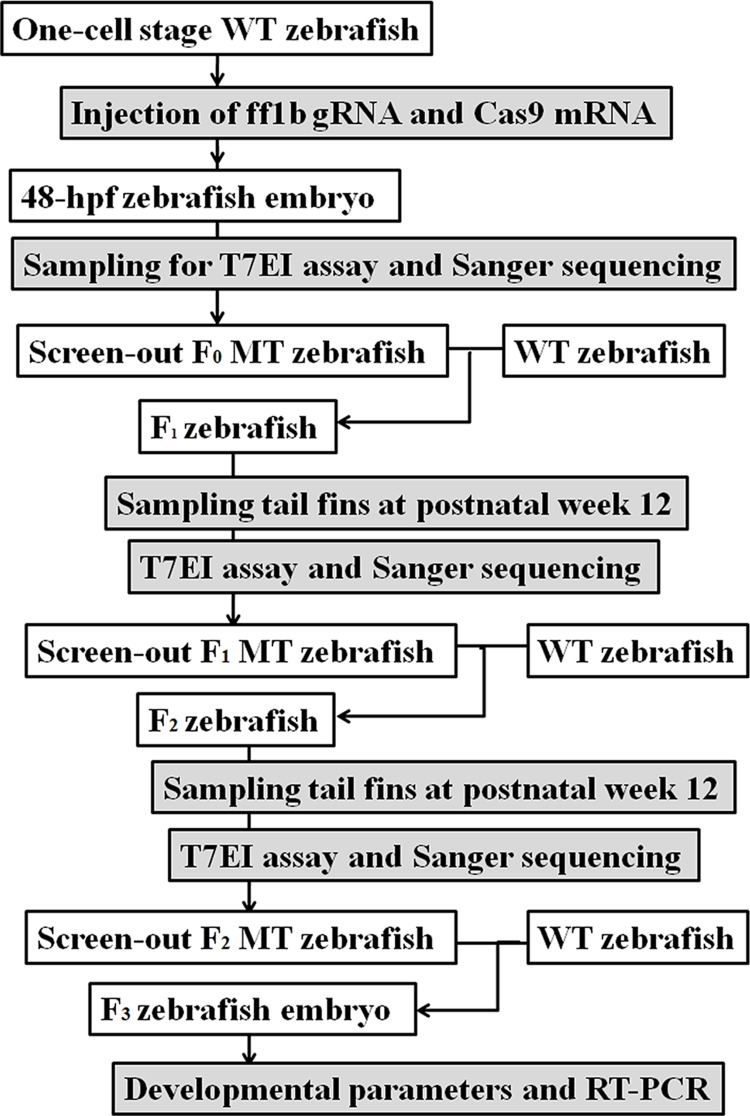
The experimental scheme for CRISPR/Cas9 targeted fushi tarazu factor 1b (ff1b) mutation in zebrafish hpf: hour post-fertilization; MT: mutant-type; RT-PCR: reverse-transcription PCR; T7EI: T7 endonuclease I; WT: wild-type.

ff1b, the SF-1 ortholog, was knocked out in zebrafish through CRISPR/Cas9 targeted mutation [47]. The experimental scheme was shown in Figure 7. The full-length Cas9 cDNA was cloned into the pXT 7 vector. The capped Cas9 mRNA was produced using the mMESSAGE mMACHINE kit. The corresponding ff1b gRNA sequence was cloned into the pMD 19-T vector, the primers of which (forward: 5'-GCGATGCTACATTCAGTGTC-3'; reverse: 5'-CCGTCAAATAAGATAACAGGG-3') were designed at http://zifit.partners.org/ZiFiT/. The gRNA sequence was amplified into double-stranded DNA by PCR. And the corresponding primers were as follows: forward: 5'-TGTAATACGACTCACTATAGAGGAGCTTTGCCCGGTGTGGTTTTAGAGCTAGA-3'; reverse: 5'-AGCACCGACTCGGTGCCACT-3'. The products were identified by agarose gel electrophoresis and purified by the TIANgel Midi purification kit.

One-cell stage zebrafish embryos were injected with 8 nL solution containing 20 ng/μL gRNA and 300 ng/μL Cas9 mRNA. Genomic DNA was isolated from these injected embryos at 48 hpf and was digested in 5 μg/mL proteinase K for 90 min at 65°C, followed by 15 min at 95°C. The genomic targeted region was amplified by PCR. Primers (annealing 58°C, 30 s) were as follows: forward: 5'-GAGCTTTCAGAGAACATTTAGAA-3'; reverse: 5'-CAGCACACCACAGTTTACTGTAG-3'. The PCR amplicon was then denatured slowly and reannealed to facilitate the heteroduplex formation. The product was digested with 5 units of T7EI at 37°C for 90 min. Then the digested samples were analyzed by agarose gel electrophoresis. For further confirmation, the purified PCR products were cloned into the pGEM-T easy vector and analyzed by Sanger sequencing.

The screen-out MT zebrafish, which were taken as the F_0_ zebrafish, were backcrossed with the WT zebrafish at postnatal week 12. At 12 hpf, dead or deformed F_1_ embryos were removed. The tail fins of F_1_ zebrafish were collected at postnatal week 12 and analyzed as described above to search for MT zebrafish. F_1_ MT zebrafish were backcrossed with the WT zebrafish at postnatal week 12. F_2_ MT zebrafish were screened out according to the screening methods in F_1_ zebrafish.

#### Development of F_3_ embryos

At postnatal week 12, F_2_ MT zebrafish were backcrossed with the WT zebrafish. At 12 hpf, dead or deformed F_3_ embryos were removed. The body length, yolk sac absorption and IUGR rate were recorded at 24 hpf, 36 hpf, 48 hpf, 72 hpf, 4dpf, 5 dpf and 6 dpf, respectively. The embryo was taken as IUGR when its body length was 10% shorter than the mean body length of the control group [46]. The body segment number was recorded at 24 hpf and 48 hpf. And embryonic development was scored at 24 hpf, 48 hpf and 72 hpf, following the GMS [48].

#### Real-time RT-PCR analysis

Real-time RT-PCR was used to detect ff1b mRNA expression in F_2_ zebrafish at postnatal week 12, and the mRNA expression of gsc and krx-20 in 12-hpf F_3_ embryos. Total RNA was isolated from the tail fins of F_2_ zebrafish and F_3_ embryos as described in *RT-PCR analysis in placentae*. Each sample was normalized on the basis of β-actin mRNA content. Primers and annealing conditions were shown in Supplementary Table 1.

### SF-1 methylation analysis in peripheral blood of pregnant women with IUGR

#### Participants

In this study, all subjects were recruited between February 2014 and May 2015 from routine prenatal screening in the Department of Obstetrics and Gynecology at Enshi General Hospital, Hubei, China. Eight Han Chinese primiparae were selected (at 28-32 gestational weeks) for normal or IUGR singleton pregnancies. IUGR was diagnosed when fetal abdominal circumference and/or estimated fetal weight were below the tenth percentile for GA, with a reduction of growth on repeated ultrasonographic evaluation and abnormal umbilical artery blood flow waveform on Doppler examination. None of the participants had histories of preexisting hypertension, diabetes mellitus, liver disease, or chronic kidney disease.

#### Peripheral blood sampling and DNA isolation

Maternal peripheral blood samples were taken by venipuncture. 3 mL of maternal blood was collected into an EDTA tube. Genomic DNA in maternal blood was extracted by the genomic DNA preparation kit and stored at -20°C for BSP analysis.

#### BSP analysis

The methylation status of CpG sites in the -483/-28 domain of SF-1 proximal promoter was determined by BSP analysis. The DNA sequence of this domain was referred to UCSC Genome Browser (http://genome.ucsc.edu/) and demonstrated in Supplementary Database 1. 5 clones were sequenced for each sample. Two primers were used for PCR amplification (SF1-1 and SF1-2), which cover the CpG rich region of proximal SF-1 promoter. Primers of SF1-1 (-483 nt ~ -190 nt) were as follows: forward: 5’-AAAAGAGGTGGAAGTAGTAGGT-3’; reverse: 5’-CCTTAACCAACTAA CTATTCCA-3’. Primers of SF1-2 (-321 nt ~ -28 nt) were as follows: forward: 5’-GGGTGGGGGAGTAGTTTATAAA-3’; reverse: 5’-CCAATTAATCTCTACCCCCAC-3’. The amplification conditions were as follows: initial denaturation at 98°C for 2 min; 45 cycles of denaturation at 95°C for 30 s, anneal at 60°C for 30 s, and extension at 72°C for 30 s; a final elongation step at 72°C for 10 min.

### Ethics statement

All procedures performed in studies involving animals were in accordance with the Guidelines for Animal Research, and were approved by the Ethical and Research Committee of Medical College of Wuhan University (No. 00092764). All procedures performed in studies involving human participants were in accordance with the ethical standards of the institutional and/or national research committee and with the 1964 Helsinki declaration and its later amendments or comparable ethical standards. The human subject research was approved by the Ethical and Research Committee of Wuhan University (No. 12016), China. The written informed consents were obtained from all participants included in the study.

### Statistical analysis

SPSS 15.0 from SPSS Science Inc. (Chicago, IL, USA) and Graphpad Prism 5.0 from GraphPad Software (La Jolla, CA, USA) were used for data analysis. All measurement data were expressed as the mean ± SD and evaluated with two-sided *t*-test. Enumeration data, such as IUGR rates were firstly calculated and then arcsine square-root transformed to make the data following normal distribution [49]. The methylation frequencies were evaluated by Fisher's exact test. Statistical significance was set at *P*<0.05.

## CONCLUSIONS

In summary, distinct DNA methylation profile exists in maternal blood of IUGR rats. SF-1 plays an important role in fetal development and the occurrence of IUGR. Hypermethylated SF-1 in the maternal rat blood could be a potential IUGR biomarker. The underlying mechanism may be as follows: hypermethylated SF-1 inhibited its gene expression in the placenta, which could lead to increased placental apoptosis and IUGR. As apoptotic cells flows into maternal blood, the changed DNA methylation patterns in apoptotic placentae can be reflected in maternal blood. To our knowledge, we firstly used genomic DNA methylation sequencing to screen out potential IUGR biomarkers in maternal blood from the perspective of DNA methylation. It will provide evidence for IUGR pathophysiology and non-invasive prenatal prediction of IUGR. Further analysis for SF-1 hypermethylation in more types of tissues on a larger scale will be needed to confirm the role of hypermethylated SF-1 in prenatal IUGR diagnosis.

## SUPPLEMENTARY MATERIALS FIGURES AND TABLES





## Abbreviations

BMI: body mass index
BSP: bisulfite sequencing PCR
CDX2: caudal type homeo box 2
CRISPR/Cas9: clustered regularly interspaced short palindromic repeats/CRISPR-associated protein 9
CRY2: cryptochrome circadian clock 2
DMR: differentially methylated region
dpf: day post-fertilization
ff1b: fushi tarazu factor 1b
GA: gestational age
GD: gestational day
GMS: general morphology score
gsc: goosecoid
HE: hematoxylin and eosin
hpf: hour post-fertilization
HSD17B2: 17-beta hydroxysteroid dehydrogenase type 2
IUGR: intrauterine growth retardation
MT: mutant-type
RARA: retinoic acid receptor alpha
RT-PCR: reverse-transcription PCR
SF-1: steroidogenic factor-1
T7EI: T7 endonuclease I
TEM: transmission electron microscope
TUNEL: TdT-mediated dUTP nick end labelling
WT: wild-type
