# Supplementary material for: Steroidogenic factor-1 hypermethylation in maternal rat blood could serve as a biomarker for intrauterine growth retardation
Source: Oncotarget. 2017 Oct 10;8(56):96139–53. doi: 10.18632/oncotarget.21767 (PMC5707087; doi:10.18632/oncotarget.21767)
Supplement: Supplementary file 1 [file oncotarget-08-96139-s001.pdf]

Steroidogenic factor-1 hypermethylation in maternal rat blood could serve as a biomarker for intrauterine growth retardation

SUPPLEMENTARY MATERIALS

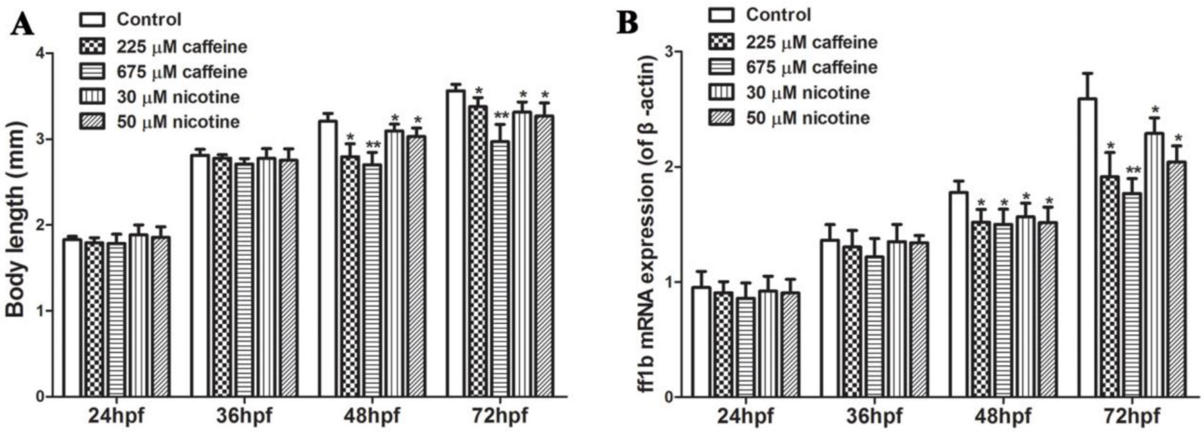

Supplementary Figure 1: Body length and fushi tarazu factor 1b (ff1b) expression in zebrafish embryos exposed to caffeine or nicotine. Real-time reverse-transcription PCR (RT-PCR) was used to detect ff1b mRNA expression. **(A)** Body length; **(B)** ff1b mRNA expression. hpf: hour post-fertilization. Mean ± SD, $^{*}P<0.05$, $^{**}P<0.01$, two-sided *t*-test, *n* = 10 zebrafish embryos.

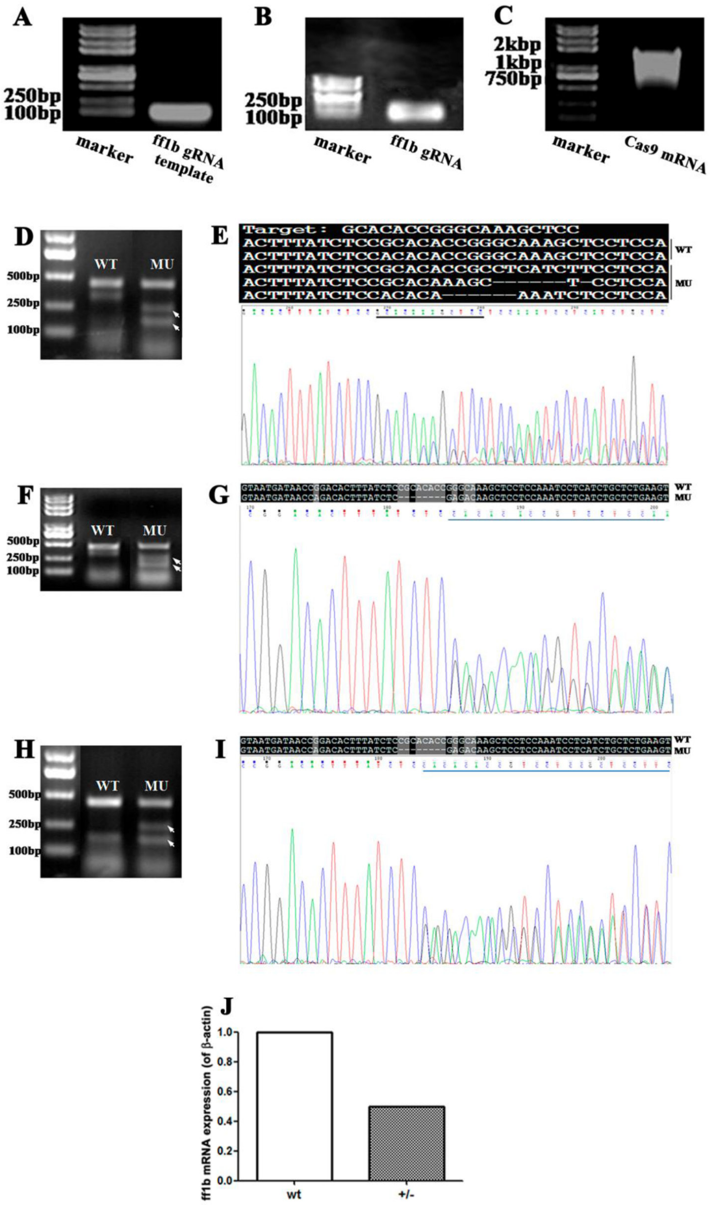

Supplementary Figure 2: CRISPR/Cas9 targeted fushi tarazu factor 1b (ff1b) mutation in zebrafish. ff1b gRNA and Cas9 mRNA of zebrafish were synthesized *in vitro*, and were injected into F_0 embryos at the one-cell stage. Genomic DNA was isolated from F_0 embryos at 48 hours post-fertilization (hpf), F_1 and F_2 zebrafish at postnatal week 12. The genomic targeted region was amplified by PCR. The product was subject to T7 endonuclease I (T7EI) assay and Sanger sequencing, respectively. **(A)** ff1b gRNA template; **(B)** ff1b gRNA; **(C)** Cas9 mRNA; **(D)** the F_0 embryos analyzed by T7EI assay; **(E)** the F_0 mutant-type (MT) embryo verified by Sanger sequencing. The line on the top of the figure indicated the mutant region. **(F)** The F_1 zebrafish analyzed by T7EI assay; **(G)** the F_1 MT zebrafish verified by Sanger sequencing; **(H)** the F_2 zebrafish analyzed by T7EI assay; **(I)** the F_2 MT zebrafish verified by Sanger sequencing. WT: wild type; MT: mutant type. White arrows: MT was digested into two bands by T7EI assay. **(J)** ff1b mRNA expression in F_2 zebrafish detected by real-time reverse-transcription PCR (RT-PCR). Mean ± SD, $^{*}P$<0.05, two-sided *t*-test, *n* = 3 embryos.

Supplementary Table 1: Oligonucleotide primers and PCR conditions of zebrafish in real-time RT-PCR

Genes	Primers (5'- 3')	Product (bp)	Annealing
ff1b	F: TCATTCCCAGCACAGTAT<br>R: TGAGTAACCCAGCGTAGA	108	60 °C, 30 s
gsc	F: GCTTATTTCTCCAGTCCCACATCAA<br>R: CGTCAGGGTATTTCGTTT	175	62 °C, 30 s
krx-20	F: AAAACTGGGTGGACTGATG<br>R: AACGAAAGGCACGACGAT	333	63 °C, 30 s
β-actin	F: CGAGCAGGAGATGGGAACC<br>R: CAACGGAAACGCTCATTGC	102	60 °C, 30 s

ff1b, fushitarazu factor 1b; gsc, goosecoid; RT-PCR, reverse-transcription PCR.

Supplementary Data 1: DNA sequence of the steroidogenic factor-1 (SF-1) proximal promoter regions for bisulfite sequencing PCR (BSP) analysis.

See Supplementary File 1
