# Supplementary material for: Steroidogenic factor-1 hypermethylation in maternal rat blood could serve as a biomarker for intrauterine growth retardation
Source: Oncotarget. 2017 Oct 10;8(56):96139–53. doi: 10.18632/oncotarget.21767 (PMC5707087; doi:10.18632/oncotarget.21767)
Supplement: Supplementary file 2 [file oncotarget-08-96139-s002.doc]

**Supplementary Data 1: DNA sequence of the steroidogenic factor-1 (SF-1) proximal promoter regions for bisulfite sequencing PCR (BSP) analysis.**

The CpG sites are in red. The transcription start site (+1) is underlined.

1. Rat SF-1 (-280 nt ~ +60 nt):

ATCTGATTTTCTCAGAATCGGGGTTTTGTTCTCAGACAAACGAATCGGGATGGAAATGCATCGAATCCGAGGGTCCCGAATCGGGCGCGGCAGAGGCGGCAAGGAAGCATCCCTGGAGCGCGGCGGGTGGCTGAGCTGCAGCGCCGGGACGCGGCCCGCAAACCCCCGCACCCGGCCGGTGCTGGGGACCGCCCGGTGTACACAGACCAGGGCAACCCCAGGCCAGTCGCCGTTGGCCCGCGCTGACTCGATCCTCCTTTCACAGGCGGACGCCGCGGGCATGGACTATTCGTACGACGAGGACCTGGACGAGCTGTGTCCAGTGTGTGGTGACAAGGTG

1. Human SF-1 (-483 nt ~ -28 nt):

AAAAGAGGTGGAAGCAGCAGGCCGCCTAGTCAAGTCGCCATGGCCTTGCCTCCGCACTGGCCTGTCCTGACTCTACTCCAATGTCCGGGCTGGGCCAGACCATGCCAGGCCAGGCCCCAGGGAGGTAGCCATTCACAAGGAGAGAGGAGCAGGCGGCAGGCCGGGTGGGGGAGCAGCCCATAAATCAAGCCCCACTCCCACCCGGTTTCTAACAAGCGGGGCTGCCTACCCGCCTCTGTGGGGTCCCTGCCTCTGGGCTCCCACAGCGGGCCTGGAACAGCCAGCTGGCCAAGGCCTCTGCAGTGCCTTGGCCTCCGCCCCCACCCTCAGCCCCCAGATAGATAGGGGTATTTTTTTTCTTTTAGGAGAAGAAGAAAAAATAGACGTAAATGAAGAGAAACACCAACAAAGAAGGCGAGAGGCCTGCAGAGTCACGTGGGGGCAGAGACCAATTGG
